# Elevation and cholera: an epidemiological spatial analysis of the cholera epidemic in Harare, Zimbabwe, 2008-2009

**DOI:** 10.1186/1471-2458-12-442

**Published:** 2012-06-18

**Authors:** Miguel A Luque Fernandez, Michael Schomaker, Peter R Mason, Jean F Fesselet, Yves Baudot, Andrew Boulle, Peter Maes

**Affiliations:** 1 Centre of Infectious Disease Epidemiology and Research (CIDER), University of Cape Town, Cape Town, South Africa; 2, Biomedical Research & Training Institute and the University of Zimbabwe College of Health Sciences, Harare, Zimbabwe; 3 Medecins sans Frontieres / Public health department: Water, Hygiene and Sanitation Unit, Amsterdam Operational Centre, Amsterdam, Netherlands; 4 NADAR sprl, Geographic Information Systems, Marchin, Belgium; 5 Medecins sans Frontieres / Medical department: Water, Hygiene and Sanitation Unit, Brussels Operational Centre, Brussel, Belgium

## Abstract

**Background:**

In highly populated African urban areas where access to clean water is a challenge, water source contamination is one of the most cited risk factors in a cholera epidemic. During the rainy season, where there is either no sewage disposal or working sewer system, runoff of rains follows the slopes and gets into the lower parts of towns where shallow wells could easily become contaminated by excretes. In cholera endemic areas, spatial information about topographical elevation could help to guide preventive interventions. This study aims to analyze the association between topographic elevation and the distribution of cholera cases in Harare during the cholera epidemic in 2008 and 2009.

**Methods:**

We developed an ecological study using secondary data. First, we described attack rates by suburb and then calculated rate ratios using whole Harare as reference. We illustrated the average elevation and cholera cases by suburbs using geographical information. Finally, we estimated a generalized linear mixed model (under the assumption of a Poisson distribution) with an Empirical Bayesian approach to model the relation between the risk of cholera and the elevation in meters in Harare. We used a random intercept to allow for spatial correlation of neighboring suburbs.

**Results:**

This study identifies a spatial pattern of the distribution of cholera cases in the Harare epidemic, characterized by a lower cholera risk in the highest elevation suburbs of Harare. The generalized linear mixed model showed that for each 100 meters of increase in the topographical elevation, the cholera risk was 30% lower with a rate ratio of 0.70 (95% confidence interval=0.66-0.76). Sensitivity analysis confirmed the risk reduction with an overall estimate of the rate ratio between 20% and 40%.

**Conclusion:**

This study highlights the importance of considering topographical elevation as a geographical and environmental risk factor in order to plan cholera preventive activities linked with water and sanitation in endemic areas. Furthermore, elevation information, among other risk factors, could help to spatially orientate cholera control interventions during an epidemic.

## Background

On the 20th of August 2008, an outbreak of 118 cases was declared at St. Mary’s and Zenenga wards of Chitungwiza, a large urban centre on the outskirts of Harare [1,2]. Vibrio Cholerae El Tor 01 was isolated from 18 (30%) of the 59 specimens submitted for examination, thus supporting the clinical evidence for an outbreak [3].

Following this initial outbreak in Chitungwiza, a second wave of infections was reported a few months later with numerous wards being affected and a rapid transmission of the infections to the whole city of Harare. This is one of the largest and most extensive outbreaks of cholera yet recorded in Zimbabwe affecting rural and urban areas [1-4].

In developing countries, cholera is closely related to poor environmental status and lack of basic infrastructure. In this respect, high population densities and poor access to safe water and proper sanitation, along with other environmental conditions, contribute to the spread of cholera in Africa [5-7]. There have been several environmental risk factors described, related to the origin of a cholera epidemic, such as sea surface temperature and more recently, air temperature and rainfall [8-15]. In highly populated African urban areas where the access to clean water is a challenge, water source contamination is one of the most cited risk factors in a cholera epidemic. During the rainy season, where there is either no sewage disposal or working sewer system, runoff of rains follows the slopes and gets into the lower parts of towns where shallow wells could easily become contaminated by excretes. In cholera endemic areas, spatial information about topographical elevation could help to guide preventive interventions.

In 1852, Farr hypothesized a causal relation between the cholera cases in the London epidemic and the elevation, indicating that there was an underlying “natural law” correlating infection with cholera inversely to elevation above high water. Farr stated that the elevation of the soil in London had a more constant relation with mortality from cholera than any other known element. In the same way, his contemporaneous colleague Snow, by mapping cholera deaths by elevation, found a correlation between higher elevations and reduced risk for cholera death [16,17].

Since 1852 there is some evidence about the association between cholera and elevation, although there is no empirical data explaining this pattern [18-20]. Cholera appearance and epidemic magnitude are related to the local environment [21]. New findings explaining possible local ecological and environmental risk factors of cholera are of importance as they could be used to prevent and plan future cholera epidemic responses in endemic areas. Therefore, this study aims to analyze the association between topographic elevation and the distribution of cholera cases, in Harare during the cholera epidemic in 2008 and 2009.

## Methods

We developed an ecological study using secondary data.

Data were drawn from the registry of cholera treatment centers (CTCs) and oral rehydration points (ORP) functioning during the cholera epidemic in Harare and Chitungwiza. Medecins Sans Frontieres, in collaboration with the Department of City Health of the Ministry of Health & Child Welfare, implemented and managed three CTCs, in the Budiriro Polyclinic, in the Beatrice Road Infectious Diseases Hospital and in the city of Chitungwiza. Ten ORPs were functioning in Harare city and one in Chitungwiza.

Data used in this study were the aggregated number of cholera cases by suburb of residence, topographic average elevation of each suburb in meters, and the distance in meters from suburbs’ geometric centre to Chitungwiza (the epicenter of the epidemic).

Population figures by suburb were calculated from the official census of Harare and Chitungwiza, completed in 2002. To estimate the populations’ figures at the time of the epidemic, we employed an average constant annual growth rate of 3%, as estimated by the Population Division of the Department of Economic and Social Affairs of the United Nations Secretariat [22].

During the epidemic, a cholera case was defined as any patient presenting 3 or more liquid stools and/or vomiting for the last 24 hours [23].

We described the elevation by suburbs and distance of each suburb to the epicenter (Chitungwiza) in meters. Geographical information was integrated into the GIS database as vector polygons. Using ArcGIS ‘zonal statistics’ module, we measured for each polygon average ground elevation and the distance between each suburb centroid (geometric center of the polygon) to Chitungwiza.

To describe the outbreak, we calculated attack rates for each suburb, including 95% exact confidence intervals (CIs). We assumed that the attack rates follow a Poisson distribution [24,25]. We then estimated rate ratios and their respective 95%CIs, taking the whole Harare rate as reference.

To describe the relation between the cholera cases and elevation, the Pearson’s correlation coefficient (R) between the cholera rates and the mean elevation of each suburb was calculated. Then, a scatter plot was built to show the relation between the observed rates and the average elevation by suburb.

A generalized linear mixed model (under the assumption of a Poisson distribution) was used to model the relation between the risk of cholera by suburb and the elevation in meters of Harare: The model assumed a random intercept, with the number of cholera cases as the dependent variable, the log of the population figures by suburb as the offset, and the suburb’s average elevation as the independent variable [24,26].

We use prior knowledge about the dynamic and spread of the epidemic to allow for spatial correlation between neighboring suburbs based on the distance in meters to the epicenter (Chitungwiza) [12]. Therefore, the random intercept of the mixed model was used to account for the dependency of neighboring suburbs, overdispersion and allowing for clustered variance estimation [27]. In detail, we constructed a categorical cluster variable *Q*, based on the quintiles (_*Q*1_−_*Q*5_) of the distance of each suburb to Chitungwiza. Hence, our final model looks as follows:

(1) $$\begin{array}{lll} \ln({\text{cases}}_{\mathrm{ij}}) & =\ln({\text{population}}_{\mathrm{ij}})+\beta_{0}+\beta_{1}\; & \;\\ & \;\times ({\text{elevation in meters}}_{\mathrm{ij}})\; & \;\\ & \;+\;Q_{j}(\text{Random effect:(five clusters}))\; & \; \end{array}$$

which corresponds to

(2) $$\begin{array}{lll} \ln({\text{cases}}_{\mathrm{ij}})-\ln({\text{population}}_{\mathrm{ij}}) & =\ln\left(\frac{\text{Cases}}{\text{population}}\right)\; & \;\\ & =\beta_{0}+\beta_{1}\; & \;\\ & \;\times \text{elevation}+Q_{j}\; & \; \end{array}$$

The derived risk ratio of the model was interpreted as the cholera risk per 100 meters of increase in the elevation, taking into account overdispersion and spatial correlation of the data.

Finally, the predicted rates of cholera by suburb were derived from the model using an Empirical Bayesian estimation. This Empirical Bayesian approach consists of computing a weighted average between the raw rate for each suburb and the regional average, with weights proportional to the underlying population at risk [28]. Estimated rates were illustrated in a figure in order to show the relation between the predicted rates and the elevation in meters. Because of the random intercept, represented by the quintile variable (Q1 to Q5), predicted rates by suburbs were clustered in groups following the specification of the model.

A sensitivity analysis was developed to confirm the consistency of our findings, consisting of an iterative random selection of suburbs to build the cluster variable for the estimation of the random effect. The aim of this process was to estimate the variability of the coefficient of the elevation, taking our first estimation as reference and proving its consistency [29,30].

For the GIS component of the analysis (mean elevation and distance to the epicenter), a recent (2008) high resolution satellite image of Harare was used. In order to assure a good integration with field surveys, the satellite image was geometrically corrected to fit a set of control points surveyed with a GPS. This image was used as a spatial reference for all GIS analysis. Information from the Harare’s topographic map (Produced by the Surveyor General of Zimbabwe), was digitized on top of the satellite image using Arcview software. The elevation information was extracted from the SRTM database (a digital terrain model obtained from the Space Shuttle mission). In this database, ground elevation is provided for each 90x90 meter cell of the model. The extent of each urban district was integrated to the GIS database as vector polygons. Using ArcGIS ‘zonal statistics’ module, average ground elevation and the distance between each suburb centroid (geometric center of the polygon) to Chitungwiza were measured for each polygon.

The statistical software used for this study was Stata v.11.2 and the module ArcMAP of ArcGIS (ESRI, v. 9.2) software for spatial and geographical representation.

## Results

**Figure 1:**
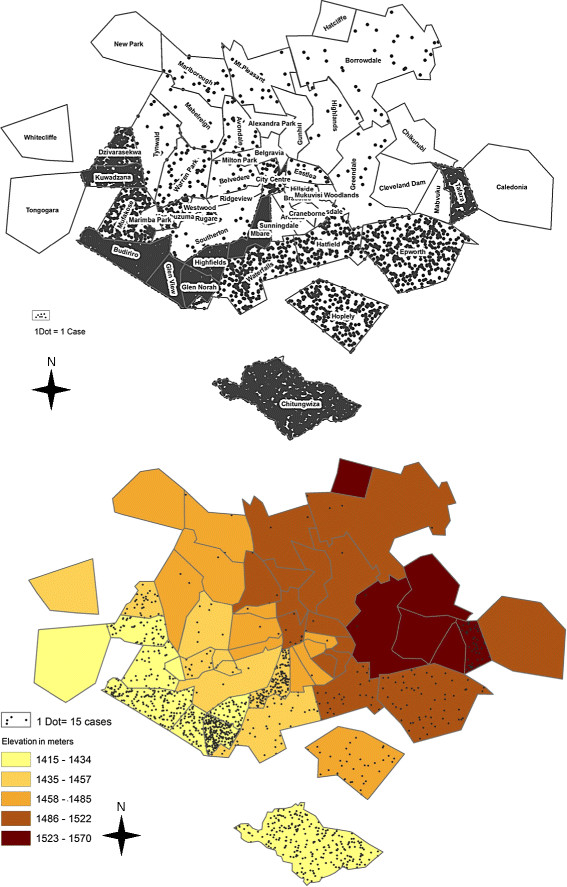
**Distribution of cholera cases and average elevation by suburb in Harare, 2008-2009 (n= 19,422 cholera cases).** (**1a**) Distribution of cholera cases by suburb (**1b**) Distribution of cholera cases and average elevation by suburb.

During this cholera outbreak, CTCs and ORPs in Harare and Chitungwiza registered and cared for 19,422 persons who met the case definition. The epidemiological description of the outbreak including the number of cases, attack rates and rate ratios by suburb is presented in Table 1. Suburbs with highest attack rates were located on the south western area of Harare, where overall, the average elevation is lower and the distance to the epicenter (Chitungwiza) is smaller (Table 1, Figure 1: 1A and 1B ).

**Table 1:** Rate ratios and attack rates per 1,000 people by suburb in Harare, 2008-2009 (n= 19,422 cholera cases)

**Suburbs**	**Average** **Elevation** **in meters**	**Distance to** **Chitungwiza** **in meters**	**Quintiles of the** **distance to Chitungwiza** **(Q1-Q5)**	**Cases**	**Population**	**Attack rates** **per 1,000 people** **(95% CI)**	**Rate ratios** **(95% CI)**
Hopley	1471	9095	1	541	5994	90.3 (82.8-98.2)	7.00 (6.43-7.63)
Waterfalls	1457	12523	1	548	7347	74.6 (68.4-81.1)	5.79 (5.32-6.30)
Mbare	1452	16562	2	2138	92219	23.2 (22.2-24.2)	1.80 (1.72-1.88)
Budiriro	1415	20484	3	2536	109545	23.2 (22.2-24.1)	1.80 (1.72-1.87)
Glen View-Glen Norah	1420	14731	1	4583	234353	19.6 (19.0-20.1)	1.52 (1.47-1.57)
Highfield	1433	15069	1	824	48713	16.9 (15.8-18.1)	1.31 (1.22-1.41)
Hatfield	1504	15857	1	355	28959	12.3 (11.0-13.6)	0.95 (0.86-1.06)
Chitungwiza	1425	0	0	3710	321782	11.5 (11.1-12.0)	0.89 (0.86-0.93)
Mufakose	1419	23106	3	568	52921	10.7 (9.8-11.6)	0.83 (0.77-0.91)
Dzivarasekwa	1440	28724	5	435	43302	10.0 (9.1-11.0)	0.78 (0.71-0.86)
Tafara	1568	24962	4	712	72737	9.8 (9.0-10.5)	0.76 (0.70-0.82)
City Centre	1489	21155	3	123	12609	9.8 (8.1-11.6)	0.76 (0.63-0.90)
Kuwadzana	1434	26513	4	759	109137	7.0 (6.4-7.4)	0.54 (0.50-0.58)
Epworth	1516	18323	2	824	130267	6.3 (5.9-6.7)	0.49 (0.46-0.53)
Kambuzuma	1439	20673	3	183	29796	6.1 (5.2-7.1)	0.48 (0.41-0.55)
Mt. Pleasant	1495	30789	5	32	5368	6.0 (4.1-8.4)	0.47 (0.33-0.65)
Malbourg	1473	32445	5	47	9600	4.9 (3.5-6.5)	0.38 (0.29-0.51)
Rugare	1445	18944	2	61	12718	4.8 (3.7-6.1)	0.37 (0.29-0.48)
Milton Park	1471	23612	4	21	4405	4.8 (2.9-7.3)	0.37 (0.24-0.57)
Avondale	1491	26243	4	47	10613	4.4 (3.2-5.8)	0.34 (0.26-0.46)
Borrowdale	1498	32400	5	63	14746	4.3 (3.2-5.4)	0.33 (0.26-0.42)
Eastlea	1495	21810	3	29	6991	4.1 (2.7-5.9)	0.32 (0.22-0.46)
Southerton	1450	17951	2	42	10385	4.0 (3.0-5.5)	0.31 (0.23-0.42)
Belvedere	1465	21532	3	30	10353	2.9 (1.9-4.1)	0.22 (0.16-0.32)
Mabelreign	1484	28900	5	16	6065	2.6 (1.5-4.2)	0.20 (0.13-0.33)
Greendale	1538	22662	3	31	14661	2.1 (1.4-3.0)	0.16 (0.12-0.23)
Arcadia	1480	17880	2	12	5681	2.1 (1.1-3.7)	0.16 (0.09-0.29)
Warren Park	1456	23702	4	100	57341	1.7 (1.4-2.1)	0.14 (0.11-0.16)
Highlands	1521	26462	4	12	6545	1.8 (0.9-3.2)	0.14 (0.08-0.25)
Hillside	1485	20116	2	8	4806	1.7 (0.7-3.2)	0.13 (0.06-0.26)
Tynwald	1460	26891	5	32	27398	1.2 (0.7-1.6)	0.09 (0.06-0.13)
All Suburbs	-	-	-	19422	1507359	12.9 (12.7-13.1)	1


This pattern of the spatial distribution of cholera cases correlates positively with the average elevation of Harare suburbs. The computed Pearson’s correlation coefficient between the cholera observed rates and the mean elevation by suburb shows a significant negative relationship, which means that at higher elevation, the number of cholera cases was lower (*R*=−0.5; p-value < 0.001).

**Figure 2:**
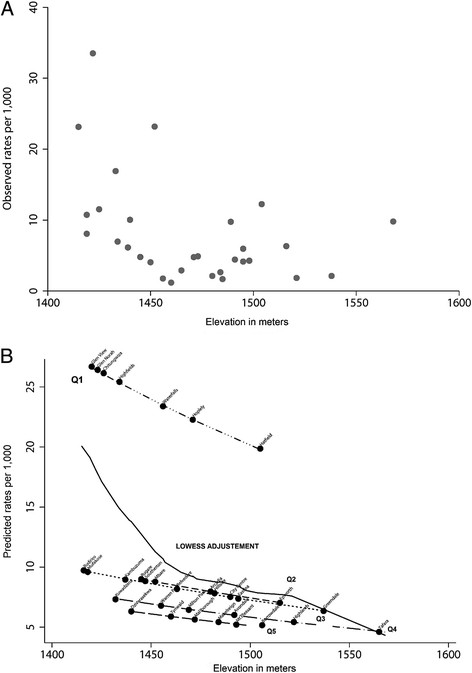
**Observed rates and predicted cholera rates based on the generalized linear mixed model by elevation and suburb in Harare, 2008-2009 (n= 19,422 cholera cases).** (**2a**) Observed cholera rates (**2b**) Predicted cholera rates based on the generalized linear mixed model. Q1-Q5 represent the quintiles relating to the distance to Chitungwiza and the random intercept in the model.

Figure 2 shows how the observed and predicted rates are related to the average elevation by suburb in Harare. To make scales of Figures 2A and 2B comparable, we exclude from Figure 2A suburbs whose rates were more than two standard deviations away from the mean attack rate (Hopley and Waterfalls). Lowess smoothing adjustment of the predicted rates (Figure 2B) in relation to the average elevation by suburbs confirms the pattern previously described in Figure 1B. The risk of cholera decreases when the average elevation increases.

Taking into account the overdispersion and the spatial correlation, the modeled relationship between the risk of cholera and the average elevation shows the same pattern (lower elevation, higher risk), with higher rate ratios for the suburbs that are located nearest to the cholera epicenter (Chitungwiza, Figure 1B and Figure 2B). In Figure 2B, Q1-Q5 represent the quintiles of the distance of each suburb to Chitungwiza; hence, Q1 refers to the cluster of suburbs nearest to the epicenter, whereas Q5 refers to the cluster of suburbs which are most far away from the epicenter. Specifically, this refers to the results of our generalized linear mixed model which showed that an increase of 100 meters in elevation yields 30% lower cholera risk, with a rate ratio of 0.70; (95%CI = 0.66-0.76). The sensitivity analysis confirmed the risk reduction with the increase of the elevation with an overall estimate between 20% and 40% (rate ratios between 0.6 and 0.8).

## Discussion

This study shows a spatial pattern of the distribution of cholera cases during the epidemic in Harare. This pattern is characterized by an increase of cholera risk for each 100 meters of drop in elevation; lowest suburbs had the highest cholera risk.

During the cholera epidemic, Zimbabwe was in economic crisis: the health care systems had become dysfunctional, water supplies were irregular and sanitation systems had collapsed. The reason for this was a lack of maintenance of the system, with frequent power interruptions affecting pumping stations [31-34]. On the 1st of December 2008, problems with the main pumping station meant that, without prior warning, the water supply was shut off for Harare city, leaving large populations without access to potable water. By 2008, Chitungwiza, a large urban centre on the outskirts of Harare, had already been without an adequate water supply water for more than 2 years. People had to revert to using unprotected shallow wells that were at risk of contamination because of the dysfunctional sewer system [35]. Unprotected shallow wells could support the pattern founded between cholera risk and elevation; hence, at lower elevation, shallow wells could be contaminated easily by sewage disposal. During the rainy season, in high populated urban areas, where there is either no sewage disposal or working sewer system, runoff of rains follows the slopes and gets into the lower parts of towns [34].

A similar pattern could have happened in Haiti where the epicenter of the epidemic had been located in the Artibonite area, which is a plain where rivers and surface water have high risk of contamination compared with mountains, situation that reinforces the external validity of our finding [36,37]. Water source contamination is one of the most cited risk factors in a cholera epidemic in all the regions of Africa; therefore, lower elevation (like plains and depressions) could facilitate contamination through surface water [38].

The diversity of regions affected by cholera epidemics during the last twenty years in Africa and the multiplicity of risk factors need to be highlighted; however, lack of water and sanitation facilities are the most common factors involved in the origin of an epidemic [38]. In any case, topographical elevation in densely populated urban areas should be considered a risk factor in endemic areas to plan cholera preventive activities linked with water and sanitation. Furthermore, elevation information could help to spatially prioritize cholera control interventions during an epidemic.

Our finding could be affected by the fact that data are based on estimated suburb population, and we recognize that the estimates may not reflect the real figures during the epidemic. However, we tried to take into account the natural average growth of the population to work with most realistic population estimates possible. The internal validity could be affected for data overdispersion and spatial correlation; however we used a mixed model with a random intercept based on a neighborhood variable which is defined via the distance to the epicenter. In introducing a random intercept, suburbs within a cluster are allowed to be dependent. Finally, to confirm whether the choice of our neighborhood cluster for the mixed effect was stable, a sensitivity analysis was developed fitting the model with many other choices of clusters specifying the dependency of suburbs (random choice, choice based on visual aspects and also a second hierarchy). Even though this methodological approach is not the classic way to account for spatial correlation, the standard errors of our estimations allow for suburbs correlation within each of the five groups (spatial correlation), relaxing the usual requirement that the observations are independent.

Our study focuses only on topographic elevation, but we have to highlight that there were other historical, geographical, social, and environmental risk factors previously published, that explained the spatial pattern of the Harare epidemic. Specifically, the fact that Tafara, a high elevated suburb, had high cholera rates, could be explained by the dynamics of population movement because there is a big concentration of bus stops and informal markets offering service to the travelers moving to Mozambique. Bus stops and informal markets, residential housing, water supplies and sewage disposal system were previously identified as risk factors related with the cholera epidemic in Harare, 2008-2009 [12]. Finally, we need to highlight that the risk of cholera by suburb cannot be attributed to one simple individual living in one specific suburb due to the ecological fallacy.

Despite that fact, and to our knowledge, this study could be the first attempt to model the relationship between cholera risk and elevation. Furthermore, the sensitivity analysis developed reinforces the validity of our final model confirming the inverse relationship between cholera risk and elevation.

## Conclusion

In conclusion, it has been shown that the distribution of cholera cases by suburb in Harare during the outbreak of 2008-2009 followed an identifiable spatial pattern characterized by a lower cholera risk when the average elevation in meters by suburb increased by 100 meters. During the rainy season, in high populated urban areas, where there is either no sewage disposal or working sewer system, runoff of rains follows the slopes and gets into the lower parts of towns. This study highlights the importance of considering topographical elevation as a geographical and environmental risk factor in order to plan cholera preventive activities linked with water and sanitation in endemic areas. Further studies are needed to explore the link between elevation, water supplies and sewage disposal systems. The identification of this spatial pattern could guide public health action to orientate further preparedness interventions in Harare, aiming to prevent a new cholera epidemic.

## Competing interests

The authors declare that they have no competing interests.

## Authors’ contributions

MALF and PM developed the concept and design of the study; JF, YB, MALF and PM acquired the data; MALF and MS developed analyses; MALF carried out the analyses, and all authors interpreted the data; MALF and MS wrote the manuscript. All authors drafted the manuscript, revised critically the content, and gave technical support and conceptual advice. Finally, all authors read and approved the final manuscript. MALF is the guarantor of the paper.

## Pre-publication history

The pre-publication history for this paper can be accessed here:

http://www.biomedcentral.com/1471-2458/12/442/prepub
